# Potential paraneoplastic syndromes and selected autoimmune conditions in patients with non-small cell lung cancer and small cell lung cancer: A population-based cohort study

**DOI:** 10.1371/journal.pone.0181564

**Published:** 2017-08-02

**Authors:** Montserrat Miret, Erzsébet Horváth-Puhó, Anouk Déruaz-Luyet, Henrik Toft Sørensen, Vera Ehrenstein

**Affiliations:** 1 Nestlé Health Science, Vevey, Switzerland; 2 Department of Clinical Epidemiology, Aarhus University Hospital, Aarhus, Denmark; 3 Boehringer Ingelheim, Global Epidemiology, Ingelheim am Rhein, Germany; University of Alabama at Birmingham, UNITED STATES

## Abstract

**Background:**

Little is known about the occurrence and distribution of types of paraneoplastic syndromes (PNS) in patients with lung cancer. Identification of autoimmune PNS is particularly important for discerning them from immune-related adverse events of novel immunotherapies. We estimated the occurrence of PNS among patients with lung cancer and compared it with that in the general population.

**Methods:**

In this registry-based cohort study in Denmark, we identified all patients with incident primary lung cancer between 1997 and 2010, and in a general-population comparison cohort matched on calendar time, sex, age, and residence. Among patients with non-small cell lung cancer (NSCLC) and small-cell lung cancer (SCLC), we estimated prevalence of potential PNS and selected autoimmune conditions and compared their incidence rates with those of equivalent conditions in the general population cohort, using hazard ratios (HRs) adjusted for baseline comorbidity.

**Results:**

There were 35,319 patients with NSCLC and 6,711 patients with SCLC. The incidence rates per 1000 person-years (95% confidence interval) of any potential PNS or selected autoimmune disorders were 135.4 (131.9–139.1) among NSCLC patients and 237.3 (224.4–250.5) among SCLC patients. Adjusted HRs for any potential PNS or selected autoimmune disorders were 4.8 (4.7–5.0) for NSCLC and 8.2 (7.6–8.8) for SCLC.

**Conclusion:**

Incidence rate of any potential PNS or selected autoimmune disorders among patients with lung cancer was greater than that in the general population and was greater after SCLC than after NSCLC.

**Impact:**

These results provide context to discerning PNS from adverse effects of novel immunotherapies during the clinical course of NSCLC and SCLC.

## Introduction

Paraneoplastic syndromes (PNS) are remote effects of cancers, unrelated to mass, invasion, or treatment [1, 2] that manifest as endocrine, neurologic, cutaneous, rheumatologic, or hematologic conditions [3]. The pathophysiology of PNS can be autoimmune (immune cross-reactivity between tumor and normal host tissues), humoral (secretion by tumors of functional peptides and hormones), or unknown [4, 5]. By definition, PNS must occur in patients with cancer; however, while detection of endocrine and hematologic PNS tends to follow a cancer diagnosis, detection of neurologic, cutaneous, and rheumatologic PNS often precedes the cancer diagnosis [3]. Period prevalence of PNS among cancer patients ranges from 7% to 15% [3, 6], but systematic population-based evidence regarding PNS occurrence is limited. Among patients with lung cancer, the reported prevalence of PNS is about 10% [7–9], based on scarce evidence, without clear reference to an underlying period or ability to distinguish between prevalent and incident cases. PNS appear to be more common in patients with small-cell lung cancer (SCLC) than in patients with non-small-cell lung cancer (NSCLC), and the two types of lung cancer may be associated with different types of PNS [7, 8, 10–13]. Approximately 9% of SCLC patients had a neurologic PNS around the time of cancer diagnosis [14]. Data on the occurrence of autoimmune conditions that are not necessarily PNS are important for providing context to immune-related adverse events of immunotherapies, which are increasingly used in cancer treatment [15, 16]. To date, no study has prospectively estimated PNS incidence rates among patients with lung cancer.

Using data linked from population-based registries in Denmark, we conducted a nationwide cohort study to estimate prevalence and incidence rates of PNS and selected autoimmune conditions among patients with NSCLC and with SCLC, as compared with the prevalence and incidence rate of the same conditions in the general population.

## Methods

### Study design and population

This cohort study was based on individual-level linkage of prospectively and routinely collected data from three nationwide registries in Denmark—the Danish Civil Registration System [17], the Danish National Patient Registry, covering all admissions to Danish somatic hospitals [18], and the Danish Cancer Registry, with mandatory reporting of all incident primary malignancies since 1987 [19]. The source population, identified from the Danish Civil Registration, included all Danish residents alive in 1997–2010. In the Danish Cancer Registry, we identified persons with incident primary lung cancer during 1997–2010 (the lung cancer cohort). Lung cancer was further classified into NSCLC and SCLC.

For each person with an incident lung cancer, we randomly sampled up to 5 persons from the general population among those who were alive on the date of the cancer diagnosis (the index date), matching on sex, year of birth, and county of residence (the general population cohort) [17].

### Endpoint ascertainment

Many well-established PNS do not have specific disease codes or may only be recognized clinically as paraneoplastic because of a proximal malignancy diagnosis. For this study based on routinely collected data on standard disease codes we therefore termed the conditions of interest as ‘potential PNS’. We defined the following categories of potential PNS: hematologic conditions; vasculitis; other vasculopathy; endocrine and metabolic conditions; neurologic conditions; conditions of the neuromuscular junction and muscle; Ménière’s disease; circulatory conditions; asthma; digestive conditions; kidney disease; dermatologic conditions; rheumatic syndromes; and non-system-specific conditions. The non-system-specific conditions category included codes for fever, cachexia, raised antibody titers, and abnormal levels of serum enzymes (acid phosphatase, alkaline phosphatase, amylase, and lipase). Among the autoimmune conditions, we included known paraneoplastic conditions as well as selected conditions not previously described as PNS (included in the categories Ménière’s disease, digestive conditions, and circulatory conditions) [20].

For both lung cancer cohort and the general population cohort, we obtained data on history of potential PNS as recorded in the Danish National Patient Registry within at least 2 years and a maximum 5 years before the index date. In most patients with PNS preceding a cancer diagnosis, the cancer is identified within one year of PNS occurrence [2].

### Follow-up for incidence rate estimates

The observation period extended from 1 January 1997 to 31 December 2011. Follow-up began on the index date and ended on the date of first-time diagnosis of a potential PNS, date of any cancer diagnosis, emigration, death, or on 31 December 2011, whichever came first. Persons in the general population cohort diagnosed with lung cancer during the follow-up were censored in the general population cohort and started contributing person-time to the lung cancer cohort. For the endpoint of any potential PNS, follow-up ended on the date of the first-recorded PNS condition. For the PNS category-specific endpoints, follow-up ended on the diagnosis date of the first condition in that category.

### Comorbidities

The following comorbidities were measured in the study population before the index date: chronic obstructive pulmonary disease (COPD), non-insulin dependent diabetes mellitus, hypertension, ischemic heart disease, and kidney disease. Furthermore, we calculated Charlson Comorbidity Index score [21], modified by excluding cancer and the comorbidities listed above, and categorized as no comorbidity [score = 0], low comorbidity [score = 1–2], and high comorbidity [score = 3+]. The comorbidities were ascertained using hospital diagnoses since 1977 [19].

### Statistical analysis

Period prevalence estimates of the potential PNS in the study population were calculated as the proportion of persons with a potential PNS diagnosed within the 2 years before the index date. Five-year prevalence was calculated in a sensitivity analysis.

The dataset for estimation of incidence rates included members of the lung cancer cohort diagnosed during 1997–2010 and matched members of the general population cohort. Members of both cohort had to be free of diagnosis of any potential PNS up to 5 years before the index date. As diagnoses made at outpatient hospital specialist clinics were available since 1995, the lookback period for outpatient diagnoses was less than 5 years patients diagnosed with lung cancer in 1997–2000. However, most prevalent PNS are expected to be detected in the 2 years preceding cancer diagnosis. All analyses were conducted separately for NSCLC and SCLC and their matched general population cohorts.

First, we tabulated distribution of age, sex, and baseline comorbidities. Then we computed incidence rates and incidence rate differences for any potential PNS and each potential PNS category and used Cox proportional-hazards regression to compute hazard ratios as estimates of the underlying relative risks of PNS for lung cancer versus the general population. The proportional-hazards assumption did not hold over the entire follow-up period, and HRs were therefore estimated separately for the first and the subsequent years of follow-up. We computed HRs adjusted for age, sex, calendar year of index date, history of COPD, non-insulin dependent diabetes mellitus, hypertension, ischemic heart disease, kidney disease, and Charlson Comorbidity Index category. Comorbidity was included to account for potential differences in rates of ascertainment of medical conditions. In secondary analyses, we stratified the analysis by age (<65, ≥65 years) and sex.

All codes for PNS used in the study are listed in the S1 Table.

This study was approved by the Danish Data Protection Agency (record number 1-16-02-1-08).

## Results

In the period 2000–2010, we identified 33,755 patients with NSCLC and 6,159 patients with SCLC in Denmark. The prevalence of any potential PNS diagnosed over the 2-year period before the index date was 11.0% in both cohorts of lung cancer patients, and the prevalence of these diagnoses was 6.0% in their corresponding matched comparison cohorts. Among patients with NSCLC, the most prevalent potential PNS at baseline were endocrine and metabolic (3.0%) conditions, neurologic (2.2%) conditions, hematologic (1.9%) conditions, and rheumatic (1.6%) conditions. Prevalence estimates observed among SCLC patients were similar: endocrine and metabolic (3.7%), neurologic (2.5%), rheumatic (1.5%), and hematologic (1.1%) conditions. The five-year prevalences were only slightly higher (data not shown).

For the analyses of PNS incidence rates, there were 35,319 patients with incident NSCLC and 6,711 patients with incident SCLC diagnosed between 1997 and 2010 and without a history of potential PNS up to 5 years before the index date. Median age at index date was 69.4 years in the NSCLC cohort and 68.2 years in the SCLC cohort. The proportion of men was 56.1% among patients with NSCLC and 54.4% among patients with SCLC. Compared with the general population, NSCLC patients were more likely to have had a history of COPD (15.1% vs 4.6%), ischemic heart disease (14.3% vs 11.2%), and kidney disease (0.9% vs 0.5%). These results were similar for SCLC (COPD [13.3% vs 4.3%], ischemic heart disease [13.5% vs 10.3%], and kidney disease [0.6% vs 0.4%]). Overall comorbidity was higher in the lung cancer cohort than in the general population cohort (Table 1).

**Table 1:** Baseline characteristics of patients with non-small cell lung cancer and small cell lung cancer cohorts and matched general population cohorts (Denmark, 1997–2010).

Characteristic	Non-small cell lung cohort		General population cohort		Small cell lung cohort		General population cohort	
	N	%	N	%	N	%	N	%
Total	35,319	100.0	150,951	100.0	6,711	100.0	29,176	100.0
Age at index date, years								
0-<55	3,802	10.8	17,926	11.9	695	10.4	3,264	11.2
55-<65	8,593	24.3	39,721	26.3	1,818	27.1	8,434	28.9
65-<75	12,320	34.9	53,491	35.4	2,664	39.7	11,644	39.9
75-<85	8,906	25.2	33,980	22.5	1,417	21.1	5,419	18.6
≥ 85	1,698	4.8	5,833	3.9	117	1.7	415	1.4
Sex								
Female	15,505	43.9	67,959	45.0	3,057	45.6	13,466	46.2
Male	19,814	56.1	82,992	55.0	3,654	54.4	15,710	53.8
Year of cancer diagnosis								
1997–2001	12,144	34.4	54,283	36.0	2,578	38.4	11,613	39.8
2002–2006	12,605	35.7	52,844	35.0	2,311	34.4	9,936	34.1
2007–2010	10,570	29.9	43,824	29.0	1,822	27.1	7,627	26.1
Chronic obstructive pulmonary disease	5,321	15.1	6,966	4.6	895	13.3	1,264	4.3
Non-insulin-dependent diabetes mellitus	1,276	3.6	5,240	3.5	261	3.9	947	3.2
Hypertension	3,863	10.9	14,869	9.9	735	11.0	2,698	9.2
Ischemic heart disease	5,048	14.3	16,865	11.2	907	13.5	3,007	10.3
Kidney disease	319	0.9	790	0.5	38	0.6	131	0.4
Charlson Comorbidity Index score								
No comorbidity: 0	25,533	72.3	124,175	82.3	4,996	74.4	24,318	83.3
Low comorbidity: 1, 2	9,176	26.0	25,333	16.8	1,621	24.2	4,607	15.8
High comorbidity: 3+	610	1.7	1,443	1.0	94	1.4	251	0.9

The overall incidence rate of any potential PNS in the NSCLC cohort was 135.4 per 1000 person-years (95% confidence interval [CI]: 131.9–139.1), and 24.2 per 1000 person-years (95% CI: 23.9–24.6) in the matched comparison cohort. In the SCLC cohort, the incidence rate of potential PNS was 237.3 per 1000 person-years (95% CI: 224.4–250.5), and 23.8 (95% CI: 23.0–24.5) in the matched comparison cohort. The highest incidence rates were observed for hematologic and non-system-specific conditions among both patients with NSCLC or SCLC, followed by endocrine and metabolic conditions (Table 2). The overall incidence rate difference for any potential PNS compared with the same conditions in the general population 111.2 per 1000 person-years (95% CI: 107.6–114.9) for NSCLC, and 213.5 per 1000 person-years (95% CI: 200.4–226.6) for SCLC (Table 3).

**Table 2:** Crude incidence rates (per 1000 person-years) for categories of potential PNS in patients with NSCLS and with SCLC and the matched general population cohorts (1997–2010).

Condition	NSCLC cohort			General population cohort for NSCLC			SCLC cohort			General population cohort for SCLC		
	Cases	Person-years	Incidence rate (95% CI)	Cases	Person-years	Incidence rate (95% CI)	Cases	Person-years	Incidence rate (95% CI)	Cases	Person-years	Incidence rate (95% CI)
All potential PNS	5,381	39,730	135.44 (131.85–139.08)	19,559	807,540	24.22 (23.88–24.56)	1,262	5,320	237.26 (224.35–250.53)	3,931	165,490	23.75 (23.02–24.50)
Hematologic conditions	2,519	43,930	57.34 (55.12–59.60)	2,729	866,380	3.15 (3.03–3.27)	682	5,860	116.37 (107.80–125.26)	528	177,830	2.97 (2.72–3.23)
Vasculitis	48	45,750	1.05 (0.77–1.37)	571	870,170	0.66 (0.60–0.71)	<5	6,500	0.62 (0.17–1.35)	118	178,600	0.66 (0.55–0.79)
Other vasculopathy	9	45,850	0.20 (0.09–0.34)	73	871,860	0.08 (0.07–0.10)	<5	6,500	0.15 (0.00–0.56)	14	178,980	0.08 (0.04–0.12)
Endocrine and metabolic conditions	988	44,550	22.18 (20.81–23.58)	5,233	856,380	6.11 (5.95–6.28)	181	6,370	28.43 (24.43–32.71)	1,046	175,690	5.95 (5.60–6.32)
Neurologic conditions	383	45,060	8.50 (7.67–9.37)	5,576	852,190	6.54 (6.37–6.72)	76	6,400	11.88 (9.36–14.70)	1,103	175,000	6.30 (5.94–6.68)
Neuromuscular junction and muscle	50	45,780	1.09 (0.81–1.42)	968	868,890	1.11 (1.04–1.19)	15	6,480	2.31 (1.29–3.62)	193	178,350	1.08 (0.93–1.24)
Ménière’s disease	9	45,840	0.20 (0.09–0.34)	206	871,190	0.24 (0.21–0.27)	<5	6,500	0.46 (0.09–1.11)	42	178,880	0.23 (0.17–0.31)
Circulatory conditions (not described as PNS)	46	45,780	1.00 (0.74–1.32)	591	870,510	0.68 (0.63–0.73)	8	6,500	1.23 (0.53–2.22)	117	178,720	0.65 (0.54–0.78)
Asthma	241	45,280	5.32 (4.67–6.01)	1,685	866,020	1.95 (1.85–2.04)	32	6,470	4.95 (3.38–6.80)	335	177,740	1.88 (1.69–2.09)
Digestive conditions (not described as PNS)	157	45,580	3.44 (2.93–4.00)	1,568	867,280	1.81 (1.72–1.90)	18	6,490	2.77 (1.64–4.19)	337	177,900	1.89 (1.70–2.10)
Kidney disease	15	45,850	0.33 (0.18–0.51)	162	871,740	0.19 (0.16–0.22)	7	6,500	1.08 (0.43–2.01)	45	178,920	0.25 (0.18–0.33)
Dermatologic conditions	73	45,690	1.60 (1.25–1.98)	744	869,660	0.86 (0.80–0.92)	5	6,500	0.77 (0.25–1.58)	162	178,530	0.91 (0.77–1.05)
Rheumatic syndromes	140	45,560	3.07 (2.59–3.60)	1,624	865,700	1.88 (1.79–1.97)	19	6,460	2.94 (1.77–4.40)	359	177,540	2.02 (1.82–2.24)
Non-system-specific conditions	1,544	44,630	34.60 (32.89–36.34)	1,064	869,700	1.22 (1.15–1.30)	449	6,140	73.08 (66.48–79.99)	228	178,540	1.28 (1.12–1.45)

CI confidence interval; NSCLC non-small cell lung cancer; SCLC small cell lung cancer

Nonzero frequencies below 5 are reported as <5 to prevent identification of individuals

**Table 3:** Incidence rate differences per 1000 person-years, and crude and adjusted hazard ratios overall and category-specific for first-time potential PNS among patients with NSCLC and with SCLC compared with general population.

	NSCLC			SCLC		
	Incidence rate difference (95% CI)	Hazard ratio (95% CI)		Incidence rate difference (95% CI)	Hazard ratio (95% CI)	
		Crude	Adjusted ^a^		Crude	Adjusted ^a^
All potential PNS	111.2 (107.6–114.9)	4.9 (4.8–5.1)	4.8 (4.7–5.0)	213.5 (200.4–226.6)	8.2 (7.7–8.8)	8.2 (7.6–8.8)
Hematologic conditions	54.2 (52.0–56.4)	14.2 (13.4–15.1)	14.1 (13.3–15.0)	113.4 (104.7–122.1)	27.7 (24.2–31.7)	27.5 (24.0–31.5)
Vasculitis	0.4 (0.1–0.7)	1.6 (1.1–2.1)	1.6 (1.2–2.1)	-0.1 (-0.7–0.6)	1.0 (0.4–2.8)	1.1 (0.4–3.0)
Other vasculopathy	0.1 (-0.0–0.2)	2.4 (1.2–4.8)	2.3 (1.1–4.6)	0.1 (-0.2–0.4)	2.3 (0.3–20.0)	1.8 (0.2–16.3)
Endocrine and metabolic conditions	16.1 (14.7–17.5)	3.4 (3.2–3.6)	3.4 (3.1–3.6)	22.5 (18.3–26.6)	4.5 (3.8–5.4)	4.8 (4.0–5.7)
Neurologic conditions	2.0 (1.1–2.8)	1.4 (1.2–1.5)	1.4 (1.3–1.6)	5.6 (2.9–8.3)	2.0 (1.6–2.5)	2.1 (1.6–2.7)
Neuromuscular junction and muscle	-0.0 (-0.3–0.3)	1.0 (0.7–1.3)	1.0 (0.8–1.3)	1.2 (0.1–2.4)	2.2 (1.3–3.9)	2.5 (1.4–4.4)
Ménière’s disease	-0.0 (-0.2–0.1)	0.7 (0.4–1.4)	0.8 (0.4–1.5)	0.2 (-0.3–0.8)	1.8 (0.5–6.3)	2.0 (0.6–6.8)
Circulatory conditions (not described as PNS)	0.3 (0.0–0.6)	1.5 (1.1–2.0)	1.3 (1.0–1.8)	0.6 (-0.3–1.4)	1.8 (0.9–3.9)	1.8 (0.8–3.8)
Asthma	3.4 (2.7–4.1)	2.4 (2.1–2.8)	1.7 (1.5–2.0)	3.1 (1.3–4.8)	1.8 (1.3–2.7)	1.3 (0.9–1.9)
Digestive conditions (not described as PNS)	1.6 (1.1–2.2)	1.9 (1.6–2.2)	1.8 (1.5–2.1)	0.9 (-0.4–2.2)	1.3 (0.8–2.1)	1.2 (0.7–2.0)
Kidney disease	0.1 (-0.0–0.3)	1.6 (0.9–2.7)	1.4 (0.8–2.5)	0.8 (0.0–1.6)	4.0 (1.7–9.4)	4.6 (1.9–11.0)
Dermatologic conditions	0.7 (0.4–1.1)	1.8 (1.4–2.3)	1.7 (1.3–2.2)	-0.1 (-0.8–0.6)	0.9 (0.4–2.2)	0.9 (0.3–2.2)
Rheumatic syndromes	1.2 (0.7–1.7)	1.5 (1.2–1.8)	1.4 (1.2–1.7)	0.9 (-0.4–2.3)	1.2 (0.8–2.0)	1.1 (0.7–1.8)
Non-system-specific	33.4 (31.7–35.1)	21.7 (19.9–23.6)	20.8 (19.1–22.7)	71.8 (65.0–78.6)	39.3 (32.7–47.4)	38.2 (31.7–46.2)

^a^ Adjusted for age, sex, residence, calendar period and baseline history of chronic obstructive pulmonary disease, non-insulin dependent diabetes mellitus, hypertension, ischemic heart disease, kidney disease, Charlson Comorbidity Index score (excluding the conditions listed beforehand).

CI confidence interval; NSCLC non-small cell lung cancer; PNS paraneoplastic syndrome(s); SCLC small cell lung cancer

The adjusted HR for any potential PNS were 4.8 (95% CI: 4.7–5.0) for NSCLC patients and 8.2 (95% CI: 7.6–8.8) for SCLC patients (Table 3), compared with the general population. Among the NSCLC patients, the HR was higher than 10 for anemia, thrombocytopenia, agranulocytosis, carcinoid syndrome, hypercalcemia, fever, and cachexia. Among the SCLC patients, the highest HRs were observed for anemia, thrombocytopenia, agranulocytosis, pituitary gland disorders—primarily accounted for by cases with the syndrome of inappropriate antidiuretic hormone secretion (SIADH), hypokalemia, ataxia, fever, and cachexia (Table 4). An increased incidence of dermatomyositis and polymyositis was observed among the SCLC patients. HRs of autoimmune conditions, especially those not previously described as paraneoplastic, were only moderately or not increased (Table 4).

**Table 4:** Hazard ratios for subcategories of first-time potential PNS among patients with NSCLC and with SCLC compared with general population.

Condition	NSCLC cohort		General population cohort	Hazard ratio (95% CI)		SCLC cohort		General population cohort	Hazard ratio (95% CI)		
	Cases	Incidence rate per 1000 person-years	Incidence rate per 1000 person-years	Crude	Adjusted ^a^	Cases	Incidence rate per 1000 person-years	Incidence Rate per 1000 person-years		Crude	Adjusted ^a^
Monoclonal proteins	9	0.20 (0.09–0.34)	0.15 (0.13–0.18)	1.3 (0.7–2.6)	1.3 (0.7–2.7)	0	-	0.19 (0.13–0.26)		-	-
‘Cytokine-mediated’	38	0.83 (0.59–1.11)	0.18 (0.15–0.21)	4.2 (2.9–6.1)	3.6 (2.4–5.2)	13	2.00 (1.06–3.23)	0.20 (0.14–0.27)		8.0 (3.9–16.5)	7.0 (3.3–14.6)
Hemolytic Uremic Syndrome and thrombotic thrombocytopenic purpura	5	0.11 (0.04–0.22)	0.02 (0.01–0.03)	5.5 (1.9–15.5)	6.5 (2.3–18.4)	0	-	0.01 (0.00–0.03)		-	-
Anemia	1,644	36.78 (35.02–38.58)	0.70 (0.64–0.75)	35.6 (32.3–39.3)	35.7 (32.3–39.4)	430	70.60 (64.08–77.42)	0.55 (0.45–0.67)		74.6 (58.4–95.3)	74.1 (57.8–94.9)
Disseminated intravascular coagulation	<5	0.07 (0.01–0.16)	0.07 (0.05–0.09)	0.9 (0.3–3.0)	0.9 (0.3–2.9)	<5	0.15 (0.00–0.56)	0.04 (0.02–0.08)		3.4 (0.4–33.1)	4.0 (0.4–38.7)
Thrombocytopenia	148	3.24 (2.74–3.78)	0.21 (0.18–0.24)	12.4 (9.9–15.7)	12.8 (10.1–16.2)	108	16.95 (13.90–20.29)	0.23 (0.17–0.31)		41.5 (27.6–62.4)	40.0 (26.6–60.2)
Agranulocytosis	171	3.74 (3.20–4.32)	0.03 (0.02–0.04)	80.9 (52.1–125.5)	71.4 (45.9–111.2)	143	22.39 (18.87–26.20)	0.04 (0.02–0.07)		229.3 (104.1–505.1)	222.8 (100.9–492.1)
Acquired coagulation factor deficiency	<5	0.09 (0.02–0.19)	0.06 (0.04–0.07)	1.6 (0.5–4.4)	1.3 (0.4–3.7)	0	-	0.06 (0.03–0.10)		-	-
Sarcoidosis	27	0.59 (0.39–0.83)	0.12 (0.10–0.14)	4.0 (2.5–6.2)	3.5 (2.2–5.4)	5	0.77 (0.25–1.58)	0.14 (0.09–0.20)		4.3 (1.5–12.8)	3.1 (1.0–9.4)
Hypercoagulability	635	13.98 (12.91–15.09)	1.70 (1.62–1.79)	7.3 (6.6–8.1)	7.2 (6.5–8.0)	81	12.56 (9.97–15.44)	1.58 (1.40–1.77)		7.8 (5.9–10.3)	7.9 (5.9–10.4)
Vasculitis	48	1.05 (0.77–1.37)	0.66 (0.60–0.71)	1.6 (1.1–2.1)	1.6 (1.2–2.1)	<5	0.62 (0.17–1.35)	0.66 (0.55–0.79)		1.0 (0.4–2.8)	1.1 (0.4–3.0)
Other vasculopathy	9	0.20 (0.09–0.34)	0.08 (0.07–0.10)	2.4 (1.2–4.8)	2.3 (1.1–4.6)	<5	0.15 (0.00–0.56)	0.08 (0.04–0.12)		2.3 (0.3–20.0)	1.8 (0.2–16.3)
Thyroid	146	3.20 (2.71–3.75)	1.26 (1.18–1.33)	2.3 (1.9–2.8)	2.3 (1.9–2.8)	23	3.54 (2.25–5.13)	1.38 (1.22–1.56)		2.4 (1.5–3.8)	2.4 (1.5–3.7)
Hypoparathyroidism	12	0.26 (0.14–0.43)	0.09 (0.07–0.11)	2.6 (1.4–5.0)	2.6 (1.4–5.0)	0	-	0.07 (0.04–0.12)		-	-
Insulin-dependent diabetes mellitus	377	8.30 (7.48–9.15)	2.86 (2.75–2.97)	2.7 (2.4–3.0)	2.8 (2.5–3.1)	74	11.47 (9.01–14.23)	2.71 (2.47–2.96)		4.0 (3.1–5.2)	4.6 (3.5–6.1)
Hypoglycemia/pancreas	69	1.51 (1.17–1.88)	0.63 (0.58–0.68)	2.4 (1.9–3.2)	2.6 (2.0–3.3)	10	1.54 (0.74–2.63)	0.62 (0.51–0.74)		4.3 (2.2–8.4)	5.3 (2.6–10.7)
Pituitary gland	9	0.20 (0.09–0.34)	0.05 (0.04–0.07)	4.1 (1.9–8.6)	4.4 (2.1–9.2)	13	2.00 (1.06–3.23)	0.06 (0.03–0.10)		22.4 (8.7–57.9)	21.0 (8.0–55.1)
Cushing	<5	0.04 (0.00–0.12)	0.02 (0.01–0.03)	2.1 (0.5–9.6)	2.0 (0.4–9.1)	<5	0.46 (0.09–1.11)	0.01 (0.00–0.03)		14.7 (2.1–102.5)	9.0 (1.3–63.6)
Other endocrine gland disorders	6	0.13 (0.05–0.25)	0.04 (0.03–0.05)	3.4 (1.4–8.4)	3.0 (1.2–7.6)	0	-	0.04 (0.02–0.07)		-	-
Carcinoid syndrome	66	1.45 (1.12–1.82)	0.03 (0.02–0.04)	40.1 (24.5–65.4)	37.3 (22.7–61.1)	<5	0.31 (0.03–0.86)	0.06 (0.03–0.10)		5.3 (1.0–29.0)	5.7 (1.0–31.3)
Other metabolic disorders	14	0.31 (0.17–0.48)	0.20 (0.17–0.23)	1.6 (0.9–2.8)	1.6 (0.9–2.7)	<5	0.46 (0.09–1.11)	0.18 (0.13–0.25)		2.2 (0.6–7.9)	2.1 (0.6–7.6)
Hypercalcemia, not otherwise specified	90	1.96 (1.58–2.39)	0.08 (0.06–0.10)	22.1 (15.8–30.9)	22.6 (16.0–31.9)	7	1.08 (0.43–2.01)	0.08 (0.05–0.13)		10.8 (3.7–31.3)	9.5 (3.2–28.2)
Acidosis	18	0.39 (0.23–0.59)	0.13 (0.11–0.15)	3.2 (1.9–5.4)	2.9 (1.7–5.0)	0	-	0.13 (0.08–0.19)		-	-
Hypokalemia	189	4.13 (3.57–4.74)	0.77 (0.72–0.83)	5.4 (4.5–6.4)	5.1 (4.2–6.0)	50	7.71 (5.72–9.99)	0.71 (0.59–0.84)		10.3 (7.1–15.0)	10.2 (7.0–15.0)
Hypertrophy of breast	39	0.85 (0.61–1.14)	0.38 (0.34–0.42)	2.3 (1.6–3.2)	2.0 (1.4–2.9)	5	0.77 (0.25–1.58)	0.35 (0.27–0.44)		2.7 (1.0–7.0)	2.7 (1.0–7.1)
Central nervous system	15	0.33 (0.18–0.51)	0.24 (0.21–0.28)	1.3 (0.8–2.2)	1.3 (0.8–2.2)	12	1.85 (0.95–3.03)	0.22 (0.15–0.29)		6.5 (3.2–13.4)	6.7 (3.2–14.0)
Movement disorders	15	0.33 (0.18–0.51)	0.39 (0.35–0.43)	0.9 (0.5–1.5)	0.9 (0.5–1.5)	7	1.08 (0.43–2.01)	0.33 (0.25–0.42)		3.8 (1.7–8.8)	4.3 (1.8–10.0)
Ataxia, unspecified	5	0.11 (0.04–0.22)	0.04 (0.03–0.05)	2.2 (0.8–5.9)	2.1 (0.8–5.7)	<5	0.15 (0.00–0.56)	0.02 (0.01–0.05)		15.5 (1.7–146.2)	18.0 (1.9–171.5)
Multiple sclerosis	11	0.24 (0.12–0.40)	0.11 (0.09–0.14)	1.6 (0.9–3.1)	1.7 (0.9–3.2)	5	0.77 (0.25–1.58)	0.12 (0.07–0.17)		4.5 (1.5–13.1)	4.3 (1.5–12.7)
Degenerative (neurology)	5	0.11 (0.04–0.22)	0.10 (0.08–0.12)	1.1 (0.4–2.6)	1.1 (0.4–2.7)	<5	0.46 (0.09–1.11)	0.12 (0.08–0.18)		3.9 (1.1–14.6)	4.7 (1.3–17.9)
Mononeuropathy	17	0.37 (0.22–0.57)	0.13 (0.10–0.15)	2.9 (1.7–4.9)	2.5 (1.4–4.2)	<5	0.15 (0.00–0.56)	0.13 (0.08–0.19)		1.4 (0.2–11.3)	1.3 (0.2–10.6)
Polyneuropathy	93	2.03 (1.64–2.47)	0.85 (0.79–0.92)	2.5 (2.0–3.1)	2.4 (1.9–3.0)	28	4.33 (2.87–6.07)	0.90 (0.77–1.05)		5.1 (3.3–7.9)	5.0 (3.2–7.9)
Autonomic neuropathy	7	0.15 (0.06–0.28)	0.11 (0.09–0.14)	1.3 (0.6–2.9)	1.4 (0.6–3.1)	<5	0.46 (0.09–1.11)	0.11 (0.06–0.16)		7.0 (1.9–26.1)	8.5 (2.2–32.1)
Eye (neurology)	229	5.05 (4.42–5.73)	4.67 (4.53–4.82)	1.2 (1.0–1.3)	1.3 (1.1–1.5)	19	2.93 (1.77–4.39)	4.47 (4.16–4.78)		0.7 (0.4–1.1)	0.8 (0.5–1.2)
Neuro muscular-junction	<5	0.04 (0.00–0.12)	0.05 (0.04–0.07)	0.9 (0.2–3.9)	1.0 (0.2–4.3)	<5	0.46 (0.09–1.11)	0.03 (0.01–0.07)		7.5 (1.5–37.8)	9.2 (1.8–46.4)
Muscle	48	1.05 (0.77–1.36)	1.06 (0.99–1.13)	1.0 (0.7–1.3)	1.0 (0.8–1.4)	12	1.85 (0.95–3.03)	1.04 (0.89–1.19)		2.0 (1.1–3.6)	2.2 (1.2–4.1)
Other myositis	0	-	0.01 (0.00–0.01)	-	-	0	-	0.01 (0.00–0.03)		-	-
Ménière’s disease	9	0.20 (0.09–0.34)	0.24 (0.21–0.27)	0.7 (0.4–1.4)	0.8 (0.4–1.5)	<5	0.46 (0.09–1.11)	0.23 (0.17–0.31)		1.8 (0.5–6.3)	2.0 (0.6–6.8)
Circulatory conditions (not described as PNS)	46	1.00 (0.74–1.32)	0.68 (0.63–0.73)	1.5 (1.1–2.0)	1.3 (1.0–1.8)	8	1.23 (0.53–2.22)	0.65 (0.54–0.78)		1.8 (0.9–3.9)	1.8 (0.8–3.8)
Asthma	241	5.32 (4.67–6.01)	1.95 (1.85–2.04)	2.4 (2.1–2.8)	1.7 (1.5–2.0)	32	4.95 (3.38–6.80)	1.88 (1.69–2.09)		1.8 (1.3–2.7)	1.3 (0.9–1.9)
Digestive conditions (not described as PNS)	157	3.44 (2.93–4.00)	1.81 (1.72–1.90)	1.9 (1.6–2.2)	1.8 (1.5–2.1)	18	2.77 (1.64–4.19)	1.89 (1.70–2.10)		1.3 (0.8–2.1)	1.2 (0.7–2.0)
Glomerulonephritis	6	0.13 (0.05–0.25)	0.06 (0.04–0.07)	2.1 (0.9–5.0)	2.0 (0.8–4.9)	<5	0.15 (0.00–0.56)	0.08 (0.05–0.13)		2.0 (0.2–16.7)	2.7 (0.3–22.8)
Glomerular disorders	<5	0.02 (0.00–0.08)	0.01 (0.01–0.02)	1.4 (0.2–11.4)	1.4 (0.2–11.7)	0	-	0.01 (0.00–0.02)		-	-
Other (renal)	8	0.17 (0.08–0.31)	0.11 (0.09–0.14)	1.4 (0.7–2.8)	1.2 (0.5–2.5)	6	0.92 (0.34–1.80)	0.16 (0.11–0.23)		4.9 (1.9–12.7)	5.2 (1.9–13.7)
Bullous (dermatology)	<5	0.07 (0.01–0.16)	0.12 (0.10–0.15)	0.6 (0.2–1.8)	0.6 (0.2–2.0)	0	-	0.14 (0.09–0.20)		-	-
Pruritus	<5	0.04 (0.00–0.12)	0.06 (0.05–0.08)	0.5 (0.1–2.0)	0.5 (0.1–2.1)	0	-	0.09 (0.05–0.14)		-	-
Alopecia	<5	0.02 (0.00–0.08)	0.02 (0.01–0.03)	1.4 (0.2–10.6)	1.1 (0.1–8.9)	0	-	0.02 (0.00–0.04)		-	-
Papulosquamous (dermatology)	<5	0.04 (0.00–0.12)	0.01 (0.00–0.01)	3.7 (0.7–20.5)	4.5 (0.8–25.0)	0	-	0.01 (0.00–0.03)		-	-
Seborrheic keratosis	17	0.37 (0.22–0.57)	0.18 (0.15–0.20)	2.4 (1.4–4.0)	2.3 (1.3–3.8)	<5	0.15 (0.00–0.56)	0.15 (0.10–0.21)		0.8 (0.1–6.2)	0.7 (0.1–5.6)
Dermatoses	7	0.15 (0.06–0.28)	0.03 (0.02–0.05)	4.2 (1.7–10.2)	4.1 (1.7–10.1)	<5	0.15 (0.00–0.56)	0.03 (0.01–0.07)		7.1 (0.6–81.1)	7.1 (0.6–82.1)
Psoriasis	27	0.59 (0.39–0.83)	0.33 (0.29–0.37)	1.9 (1.3–2.8)	1.6 (1.1–2.4)	<5	0.15 (0.00–0.56)	0.39 (0.30–0.48)		0.4 (0.1–3.1)	0.4 (0.1–3.0)
Erythematous (dermatology)	12	0.26 (0.14–0.43)	0.09 (0.07–0.11)	2.4 (1.3–4.5)	2.2 (1.1–4.1)	<5	0.31 (0.03–0.86)	0.07 (0.04–0.12)		3.8 (0.8–18.8)	3.5 (0.7–17.9)
Other (dermatology)	<5	0.09 (0.02–0.19)	0.04 (0.03–0.05)	2.3 (0.8–6.6)	2.6 (0.9–7.6)	0	-	0.03 (0.01–0.06)		-	-
Arthropathies	32	0.70 (0.48–0.96)	0.52 (0.47–0.56)	1.3 (0.9–1.8)	1.2 (0.8–1.7)	<5	0.62 (0.17–1.35)	0.57 (0.46–0.68)		1.0 (0.4–2.8)	0.9 (0.3–2.4)
Rheumatoid arthritis	79	1.73 (1.37–2.13)	1.04 (0.97–1.10)	1.5 (1.2–1.9)	1.5 (1.2–1.9)	11	1.70 (0.85–2.84)	1.13 (0.98–1.30)		1.3 (0.7–2.5)	1.2 (0.6–2.2)
Autoimmune syndromes (rheumatology)	34	0.74 (0.51–1.01)	0.37 (0.33–0.41)	1.7 (1.2–2.5)	1.6 (1.1–2.4)	<5	0.62 (0.17–1.35)	0.41 (0.32–0.51)		1.2 (0.4–3.4)	1.0 (0.4–2.9)
Panniculitis	0	-	0.01 (0.00–0.01)	-	-	0	-	0.01 (0.00–0.02)		-	-
Fever	1,126	25.19 (23.74–26.68)	1.06 (0.99–1.13)	18.3 (16.6–20.0)	17.6 (16.0–19.4)	381	61.94 (55.87–68.31)	1.14 (0.99–1.30)		36.7 (30.1–44.9)	35.8 (29.2–43.8)
Cachexia	441	9.63 (8.75–10.55)	0.15 (0.12–0.18)	48.3 (39.4–59.3)	45.6 (37.0–56.1)	73	11.24 (8.81–13.96)	0.12 (0.07–0.17)		73.1 (42.5–125.9)	70.7 (40.7–122.8)
Laboratory	<5	0.04 (0.00–0.12)	0.02 (0.01–0.03)	2.6 (0.6–11.8)	2.9 (0.6–13.0)	0	-	0.02 (0.01–0.05)		-	-

^a^ Adjusted for age, sex, residence, calendar period and baseline history of chronic obstructive pulmonary disease, non-insulin dependent diabetes mellitus, hypertension, ischemic heart disease, kidney disease, Charlson Comorbidity Index score (excluding the conditions listed beforehand).

CI confidence interval; NSCLC non-small cell lung cancer; PNS paraneoplastic syndrome(s); SCLC small cell lung cancer.

Nonzero frequencies below 5 are reported as <5 to prevent identification of individuals

The adjusted HR for any potential PNS among patients with NSCLC was 6.7 (95% CI: 6.4–7.1) in persons younger than 65 years and 3.8 (95% CI: 3.6–4.0) in persons 65 years old or older. The HRs did not vary by sex. Among patients with SCLC, the HR was 10.3 (95% CI: 9.1–11.5) for those younger than 65 years of age and 6.8 (95% CI: 6.2–7.5) for persons 65 years old or older. The adjusted HR was 7.9 (95% CI: 7.1–8.8) in women and 8.5 (95% CI: 7.6–9.4) in men. The HRs of all potential PNS were considerably higher during the first year following the cancer diagnosis date for both NSCLC (8.0 [95% CI: 7.6–8.4]) and SCLC patients (12.2 [95% CI: 11.1–13.5]), compared with the follow-up period beyond 1 year, 2.8 (95% CI: 2.7–3.0) for NSCLC and 4.0 (95% CI: 3.5–4.7) for SCLC patients. This association was particularly pronounced for the hematologic and non-system-specific PNS categories (Table 5).

**Table 5:** Crude and adjusted incidence rate ratios and 95% confidence intervals for categories of potential PNS comparing the NSCLC and SCLC cohorts with their respective matched comparison cohorts, stratifying on follow-up length (1997–2010).

	NSCLC				SCLC			
	Follow-up 0–1 year		Follow-up > 1 year		Follow-up 0–1 year		I Follow-up > 1 year	
	Crude IRR	Adjusted IRR ^a^	Crude IRR	Adjusted IRR ^a^	Crude IRR	Adjusted IRR ^a^	Crude IRR	Adjusted IRR ^a^
All clinical disorders that could be PNS	8.3 (8.0–8.7)	8.0 (7.6–8.4)	2.8 (2.7–3.0)	2.8 (2.7–3.0)	12.7 (11.5–14.0)	12.2 (11.1–13.5)	3.9 (3.4–4.5)	4.0 (3.5–4.7)
Hematologic conditions	35.0 (31.5–39.0)	34.4 (30.8–38.3)	6.9 (6.3–7.6)	7.0 (6.4–7.7)	68.0 (52.6–87.9)	66.3 (51.2–85.8)	11.9 (9.4–15.1)	12.5 (9.8–15.9)
Vasculitis	2.0 (1.3–3.2)	2.0 (1.3–3.2)	1.2 (0.8–1.9)	1.3 (0.8–2.0)	1.4 (0.4–4.8)	1.5 (0.4–5.3)	0.6 (0.1–4.2)	0.6 (0.1–4.4)
Other vasculopathy	2.6 (0.8–8.0)	2.3 (0.8–7.3)	2.2 (0.9–5.6)	2.2 (0.9–5.5)	5.8 (0.4–93.0)	3.7 (0.2–65.3)	-	-
Endocrine and metabolic conditions	5.2 (4.6–5.7)	5.1 (4.5–5.6)	2.3 (2.1–2.6)	2.3 (2.1–2.6)	6.9 (5.4–8.7)	6.8 (5.3–8.7)	2.6 (1.9–3.5)	2.9 (2.1–4.0)
Neurologic conditions	1.5 (1.2–1.7)	1.5 (1.2–1.7)	1.3 (1.1–1.5)	1.4 (1.2–1.6)	2.2 (1.6–3.1)	2.3 (1.6–3.1)	1.7 (1.2–2.5)	1.9 (1.3–2.8)
Neuromuscular junction and muscle	1.5 (1.0–2.1)	1.5 (1.0–2.2)	0.6 (0.4–1.0)	0.7 (0.4–1.1)	3.5 (1.7–7.3)	3.6 (1.7–7.6)	1.3 (0.5–3.5)	1.5 (0.6–4.1)
Ménière’s disease	0.8 (0.3–2.1)	0.9 (0.3–2.3)	0.6 (0.2–1.7)	0.7 (0.3–1.9)	1.9 (0.4–9.3)	2.3 (0.5–10.9)	1.7 (0.2–12.6)	1.8 (0.2–13.6)
Circulatory conditions (not described as PNS)	2.0 (1.3–3.1)	1.6 (1.0–2.6)	1.2 (0.8–1.8)	1.1 (0.7–1.7)	1.1 (0.3–3.8)	1.0 (0.3–3.5)	2.7 (1.1–6.6)	2.8 (1.1–6.9)
Asthma	3.3 (2.7–4.0)	2.3 (1.9–2.9)	1.7 (1.4–2.2)	1.2 (1.0–1.5)	2.2 (1.4–3.4)	1.5 (0.9–2.4)	1.2 (0.5–2.6)	0.8 (0.4–1.9)
Digestive conditions (not described as PNS)	2.1 (1.6–2.7)	2.0 (1.5–2.6)	1.7 (1.4–2.2)	1.7 (1.3–2.1)	1.5 (0.8–2.7)	1.3 (0.7–2.3)	1.0 (0.4–2.4)	1.0 (0.4–2.5)
Kidney disease	2.1 (1.1–4.3)	1.6 (0.8–3.4)	1.1 (0.5–2.7)	1.1 (0.4–2.7)	4.7 (1.5–14.0)	5.2 (1.7–16.3)	3.2 (0.7–13.5)	4.0 (0.9–17.2)
Dermatologic conditions	2.1 (1.5–3.0)	2.0 (1.4–2.9)	1.6 (1.2–2.3)	1.5 (1.1–2.2)	1.6 (0.6–4.2)	1.6 (0.6–4.3)	-	-
Rheumatic syndromes	2.0 (1.6–2.5)	1.8 (1.4–2.4)	1.1 (0.8–1.4)	1.0 (0.8–1.3)	1.1 (0.6–2.1)	0.9 (0.5–1.7)	1.4 (0.7–2.9)	1.3 (0.6–2.6)
Non-system-specific	50.5 (42.7–59.6)	48.5 (41.0–57.4)	12.9 (11.4–14.5)	12.7 (11.2–14.3)	85.3 (59.1–123.2)	81.3 (56.2–117.7)	22.9 (17.4–30.1)	24.1 (18.2–31.9)

^a^ Adjusted for age, sex, residence, calendar period and baseline history of chronic obstructive pulmonary disease, non-insulin dependent diabetes mellitus, hypertension, ischemic heart disease, kidney disease, Charlson Comorbidity Index score (excluding the conditions listed beforehand).

CI confidence interval; IRR incidence rate ratio; NSCLC non-small cell lung cancer; PNS paraneoplastic syndrome(s); SCLC small cell lung cancer

## Discussion

To the best of our knowledge, this is the first population-based cohort study to examine the occurrence of potential PNS and selected autoimmune conditions in patients with NSCLC and SCLC. The overall incidence rate of any potential PNS was nearly fivefold greater among patients with NSCLC and eightfold greater among patients with SCLC compared with the incidence of the same conditions in the general population. Hematologic and non-system-specific conditions drove the risk increase in both lung cancer cohorts, with anemia, thrombocytopenia, agranulocytosis, fever, cachexia being especially increased among SCLC patients.

It is known that incidence of PNS among patients with SCLC is higher than in those with NSCLC. This can be linked to cancer’s histological origin, as all SCLC are derived from neuroendocrine cells, whereas among NSCLC, only carcinoid and large-cell neuroendocrine carcinoma—both uncommon—are neuroendocrine tumors [22]. Neuroendocrine cells secrete peptide hormones, and neural antibodies are more frequently expressed in SCLC than in NSCLC [23, 24].

In the NSCLC and SCLC cohorts, the increased risk of potential PNS was greater in the first year following cancer diagnosis than in the subsequent years. The possible explanations for this finding could include greater severity of malignant disease at the time of diagnosis, time-dependent decrease in paraneoplastic effects of cancer due to treatment, survival effect, subsiding toxicity of chemotherapy, or tapering off of medical surveillance heightened immediately following cancer diagnosis. A PNS condition may not differ clinically from the equivalent condition in the absence of cancer, and may be recognized as paraneoplastic only because of a recently diagnosed malignancy. Thus, it would be expected that the risk of these conditions would be increased in persons with a known malignancy, compared with the general population. The greater risk increase observed in the first post-diagnosis year among NSCLC patients is consistent with this theory, and could be partially attributed to diagnostic bias. For all categories of conditions, we observed stronger associations in the younger age groups. This is expected because incidence rates of most conditions increase with age, implying that the background rates are lower in younger age groups.

Prevalence of neurological potential PNS among patients with SCLC was 2.5% in our study, which was lower than the prevalence reported in a prospective field study based on patients who had seen a neurologist prior to cancer diagnosis (4.5%) [14]. Similarly, the incidence of Lambert-Eaton syndrome among SCLC patients in our study (0.46 per 1000 person-years) is presumably much lower than the proportion of incident cases reported in other studies over unspecified times of follow-up (0% to 0.8%) [25–27]. Among patients with SCLC, incidence of SIADH is much lower than the proportion of patients with incident SIADH (1.7%) reported in a small study with active follow up of neurological disorders [27] and a median survival time shorter than 1 year. Carcinoid tumors represent only 1%-2% of NSCLC or SCLC cases [28, 29]. The carcinoid syndrome is thought to manifest in approximately 10% of carcinoid tumors [30], consistent with the number of carcinoid syndrome cases identified in our study. We found a seemingly higher risk of carcinoid syndrome for NSCLC than for SCLC.

This study was based on routine prospective data collection and individual-level data linkage in a setting of universal access to health care permits virtually complete lifetime follow-up of persons in Danish registry-based studies, including complete ascertainment of incident primary lung cancers and complete follow-up of patients’ hospital visits, emigration, and deaths [17]. At the same time, use of routinely registered hospital diagnoses to identify PNS presents a challenge in the absence of detailed clinical data. Most paraneoplastic syndromes do not have a specific disease code indicating their paraneoplastic status. In the current study, some events considered as potential PNS could be complications of cancer treatment or have a different etiology. For example, fever, cachexia, and anemia are common and multifactorial in cancer patients. They may represent complications of malignancy or result from chemotherapy [21, 25]. In lung cancer patients these conditions have been well described as PNS [31–33]. Many PNS cases in this study carried a diagnostic code of unspecified fever, which may represent a mixture of paraneoplastic fever and fever of unknown origin. Cachexia in cancer patients may result from both reduced food intake or from altered metabolism [34]. Anemia, the driving diagnosis behind the increased risk of hematologic PNS in our data, has been described as a common PNS in patients with untreated lung cancer, albeit with a more inclusive definition of anemia [20]. We included a restricted number of anemias, and excluded those secondary to bleeding. In our data, we could not distinguish whether fever, cachexia or anemia was a true PNS or a complication of cancer or its treatment. Making such distinction, however, is challenging not only in routine data, but also in clinical practice. In fact, with the exception of neurological PNS [35], no clinical diagnostic criteria exist to distinguish PNS from syndromes that are coincidental with cancer. Furthermore, not all potential PNS conditions are diagnosed in a hospital setting, leading to underascertainment of conditions that do not lead to a hospital encounter. At the same time, the observed increases in the incidence of potential PNS following the diagnosis of lung cancer attests indirectly to the reasonable quality of the registry data for at least some of the conditions under study. Finally, the large study size permitted identification of many relatively rare conditions, however, some estimates were still imprecise.

In this study, incidence rate of any potential PNS or selected autoimmune disorders among patients with lung cancer was greater than that in the general population and greater after SCLC than after NSCLC. These results may provide context about background occurrence of these conditions when treating patients with novel cancer therapies.

## Supporting information

S1 Table (DOCX)

## References

[1] De Vita VT, Lawrence TS, Rosenberg SA. DeVita, Hellman, and Rosenberg's cancer: principles & practice of oncology. 9th ed Philadelphia: Wolters Kluwer Health/Lippincott Williams & Wilkins; 2011.

[2] Darnell RB, Posner JB. Paraneoplastic syndromes. New York: Oxford University Press; 2011.

[3] Pelosof LC, Gerber DE. Paraneoplastic syndromes: an approach to diagnosis and treatment. Mayo Clin Proc. 2010;85(9):838–54. doi: 10.4065/mcp.2010.0099 20810794 10.4065/mcp.2010.0099 PMC2931619

[4] Bilynsky BT, Dzhus MB, Litvinyak RI. The conceptual and clinical problems of paraneoplastic syndrome in oncology and internal medicine. Exp Oncol. 2015;37(2):82–8. 26112932

[5] Santacroce L, Balducci L, Diomede L. Paraneoplastic syndromes. Medscape. 2016.

[6] Richardson GE, Johnson BE. Paraneoplastic syndromes in lung cancer. Curr Opin Oncol. 1992;4(2):323–33. 1591305 10.1097/00001622-199204000-00014

[7] Patel AM, Davila DG, Peters SG. Paraneoplastic syndromes associated with lung cancer. Mayo Clin Proc. 1993;68(3):278–87. 8474272 10.1016/s0025-6196(12)60050-0

[8] Hauber HP. [Paraneoplastic syndromes in lung cancer]. Pneumologie. 2011;65(6):347–58. doi: 10.1055/s-0030-1256118 21267813 10.1055/s-0030-1256118

[9] Spiro SG, Gould MK, Colice GL, American College of Chest P. Initial evaluation of the patient with lung cancer: symptoms, signs, laboratory tests, and paraneoplastic syndromes: ACCP evidenced-based clinical practice guidelines (2nd edition). Chest. 2007;132(3 Suppl):149S–60S. doi: 10.1378/chest.07-1358 17873166 10.1378/chest.07-1358

[10] Gandhi L, Johnson BE. Paraneoplastic syndromes associated with small cell lung cancer. J Natl Compr Canc Netw. 2006;4(6):631–8. 16813730 10.6004/jnccn.2006.0052

[11] Honnorat J, Antoine JC. Paraneoplastic neurological syndromes. Orphanet J Rare Dis. 2007;2:22 doi: 10.1186/1750-1172-2-22 17480225 10.1186/1750-1172-2-22 PMC1868710

[12] Titulaer MJ, Soffietti R, Dalmau J, Gilhus NE, Giometto B, Graus F, et al Screening for tumours in paraneoplastic syndromes: report of an EFNS task force. Eur J Neurol. 2011;18(1):19–e3. doi: 10.1111/j.1468-1331.2010.03220.x 20880069 10.1111/j.1468-1331.2010.03220.x PMC3086523

[13] Darnell RB, Posner JB. Paraneoplastic syndromes affecting the nervous system. Semin Oncol. 2006;33(3):270–98. doi: 10.1053/j.seminoncol.2006.03.008 16769417 10.1053/j.seminoncol.2006.03.008

[14] Gozzard P, Woodhall M, Chapman C, Nibber A, Waters P, Vincent A, et al Paraneoplastic neurologic disorders in small cell lung carcinoma: A prospective study. Neurology. 2015;85(3):235–9. doi: 10.1212/WNL.0000000000001721 26109714 10.1212/WNL.0000000000001721 PMC4516293

[15] Brahmer JR, Pardoll DM. Immune checkpoint inhibitors: making immunotherapy a reality for the treatment of lung cancer. Cancer Immunol Res. 2013;1(2):85–91. doi: 10.1158/2326-6066.CIR-13-0078 24777499 10.1158/2326-6066.CIR-13-0078 PMC4856021

[16] Jordan JT. Neurologic Immune-Related Adverse Events in Oncology Care. JAMA Neurol. 2016;73(8):907–8. doi: 10.1001/jamaneurol.2016.1564 27271299 10.1001/jamaneurol.2016.1564

[17] Schmidt M, Pedersen L, Sorensen HT. The Danish Civil Registration System as a tool in epidemiology. Eur J Epidemiol. 2014;29(8):541–9. doi: 10.1007/s10654-014-9930-3 24965263 10.1007/s10654-014-9930-3

[18] Schmidt M, Schmidt SA, Sandegaard JL, Ehrenstein V, Pedersen L, Sorensen HT. The Danish National Patient Registry: a review of content, data quality, and research potential. Clin Epidemiol. 2015;7:449–90. doi: 10.2147/CLEP.S91125 26604824 10.2147/CLEP.S91125 PMC4655913

[19] Gjerstorff ML. The Danish Cancer Registry. Scand J Public Health. 2011;39(7 Suppl):42–5. doi: 10.1177/1403494810393562 21775350 10.1177/1403494810393562

[20] Lahita RG, Chiorazzi N, Reeves WH. Textbook of the Autoimmune Diseases. Philadelphia: Lippincott Williams & Wilkins; 2000.

[21] Thygesen SK, Christiansen CF, Christensen S, Lash TL, Sorensen HT. The predictive value of ICD-10 diagnostic coding used to assess Charlson comorbidity index conditions in the population-based Danish National Registry of Patients. BMC Med Res Methodol. 2011;11:83 doi: 10.1186/1471-2288-11-83 21619668 10.1186/1471-2288-11-83 PMC3125388

[22] Travis WD. Advances in neuroendocrine lung tumors. Ann Oncol. 2010;21 Suppl 7:vii65–71. 20943645 10.1093/annonc/mdq380

[23] Schleusener JT, Tazelaar HD, Jung SH, Cha SS, Cera PJ, Myers JL, et al Neuroendocrine differentiation is an independent prognostic factor in chemotherapy-treated nonsmall cell lung carcinoma. Cancer. 1996;77(7):1284–91. doi: 10.1002/(SICI)1097-0142(19960401)77:7<1284::AID-CNCR9>3.0.CO;2-I 8608504 10.1002/(SICI)1097-0142(19960401)77:7<1284::AID-CNCR9>3.0.CO;2-I

[24] Howe MC, Chapman A, Kerr K, Dougal M, Anderson H, Hasleton PS. Neuroendocrine differentiation in non-small cell lung cancer and its relation to prognosis and therapy. Histopathology. 2005;46(2):195–201. doi: 10.1111/j.1365-2559.2005.02047.x 15693892 10.1111/j.1365-2559.2005.02047.x

[25] van Oosterhout AG, van de Pol M, ten Velde GP, Twijnstra A. Neurologic disorders in 203 consecutive patients with small cell lung cancer. Results of a longitudinal study. Cancer. 1996;77(8):1434–41. doi: 10.1002/(SICI)1097-0142(19960415)77:8<1434::AID-CNCR3>3.0.CO;2-C 8608526 10.1002/(SICI)1097-0142(19960415)77:8<1434::AID-CNCR3>3.0.CO;2-C

[26] Sculier JP, Feld R, Evans WK, DeBoer G, Shepherd FA, Payne DG, et al Neurologic disorders in patients with small cell lung cancer. Cancer. 1987;60(9):2275–83. 2830955 10.1002/1097-0142(19871101)60:9<2275::aid-cncr2820600929>3.0.co;2-3

[27] Seute T, Leffers P, ten Velde GP, Twijnstra A. Neurologic disorders in 432 consecutive patients with small cell lung carcinoma. Cancer. 2004;100(4):801–6. doi: 10.1002/cncr.20043 14770437 10.1002/cncr.20043

[28] Bertino EM, Confer PD, Colonna JE, Ross P, Otterson GA. Pulmonary neuroendocrine/carcinoid tumors: a review article. Cancer. 2009;115(19):4434–41. doi: 10.1002/cncr.24498 19562772 10.1002/cncr.24498

[29] Beasley MB, Brambilla E, Travis WD. The 2004 World Health Organization classification of lung tumors. Semin Roentgenol. 2005;40(2):90–7. 15898407 10.1053/j.ro.2005.01.001

[30] Bishop AE, Hammond PJ, Polak JM, Bloom SR. Hormones and the gastrointestinal tract In: Warrell DA, Cox TM, Firth JD, editors. Oxford textbook of medicine. 5th ed Oxford, UK: Oxford University Press; 2010.

[31] Heinemann S, Zabel P, Hauber H. Paraneoplastic syndromes in lung cancer. Cancer Therapy. 2008;6:687–98.

[32] Suzuki H, Asakawa A, Amitani H, Nakamura N, Inui A. Cancer cachexia-pathophysiology and management. J Gastroenterol. 2013;48(5):574–94. doi: 10.1007/s00535-013-0787-0 23512346 10.1007/s00535-013-0787-0 PMC3698426

[33] Zell JA, Chang JC. Neoplastic fever: a neglected paraneoplastic syndrome. Support Care Cancer. 2005;13(11):870–7. doi: 10.1007/s00520-005-0825-4 15864658 10.1007/s00520-005-0825-4

[34] Tisdale MJ. Mechanisms of cancer cachexia. Physiol Rev. 2009;89(2):381–410. doi: 10.1152/physrev.00016.2008 19342610 10.1152/physrev.00016.2008

[35] Graus F, Delattre JY, Antoine JC, Dalmau J, Giometto B, Grisold W, et al Recommended diagnostic criteria for paraneoplastic neurological syndromes. J Neurol Neurosurg Psychiatry. 2004;75(8):1135–40. doi: 10.1136/jnnp.2003.034447 15258215 10.1136/jnnp.2003.034447 PMC1739186

