# Supplementary material for: Potential paraneoplastic syndromes and selected autoimmune conditions in patients with non-small cell lung cancer and small cell lung cancer: A population-based cohort study
Source: PLoS One. 2017 Aug 2;12(8):e0181564. doi: 10.1371/journal.pone.0181564 (PMC5540596; doi:10.1371/journal.pone.0181564)
Supplement: S1 Table — (DOCX) [file pone.0181564.s001.docx]

# S1 Table: List of the ICD-10 codes used to identify cases in each category and sub-category of PNS and selected autoimmune conditions.

|  | **ICD-10 codes** |
| --- | --- |
| **Potential PNS** | |
| **Hematologic conditions** |  |
| Monoclonal proteins | D47.2, D89.1, E85.3, E85.4, E85.8, E85.9 |
| H-E (‘Cytokine-mediated’) | D47.3, D72.1, D72.8, D75.1 |
| Hemolytic Uremic Syndrome and TTP | D59.3, M31.1 |
| Anemia | D51.0, D59.1, D59.4, D59.9, D60, D61.3, D61.9, D63.0, D64.8 |
| Disseminated intravascular coagulation | D65 |
| H-thrombocytopenia | D69.3, D69.5, D69.6 |
| Agranulocytosis | D70 |
| Acquired coagulation factor deficiency | D68.4 |
| Sarcoidosis | D86 |
| Hypercoagulability | I74.9, D68.6, I80.2, I26, I82.1 |
| **Vasculitis** | D69.0, I67.7, L95, I77.6, M30.0, M30.1, M31.0, M31.3, M31.5, M31.6, M31.7 |
| **Vasculopathy** | I73.0, I73.1, I73.8 |
| **Endocrine and metabolic conditions** |  |
| E-thyroid | E05.0, E05.1, E05.2, E06, E06.1, E06.3, E06.4, E06.9, E07.0, E07.8, E07.9 |
| Hypoparathyroidism | E20 |
| Insulin-dependent diabetes mellitus | E10 |
| E-hypoglycemia/pancreas | E15, E16.1, E16.2, E16.4 |
| E-pituitary gland | E22.0, E22.2, E23.0, E23.7 |
| E-Cushing | E24.0, E24.3, E24.8, E24.9 |
| E-other endocrine gland disorders | E27.1, E28.3, E31.0, E32 |
| Carcinoid syndrome | E34.0 |
| E-other metabolic disorders | E83.1, E83.3, M83, E87.0 |
| Hypercalcemia NOS | E83.5C |
| Acidosis | E87.2 |
| Hypokalaemia | E87.6 |
| Hypertrophy of breast | N62 |
| **Neurologic conditions** |  |
| N-CNS | G04.0, G04.8, G04.9, G05.8*, G99.2*, G12.2, G13.1*, G36.0, G36.1, G93.4, G94.8* |
| N-movement disorders | G21.8, G21.9, G22*, G25.3, G25.8, G25.5, G25.9, R25.1 |
| Ataxia, unspecified | R27.0 |
| Multiple sclerosis | G35 |
| N-degenerative | G31.9, G32.8* |
| N-mononeuropathy | G56.4, G58.7, G56.9, G57.9, G58.9 |
| N-polyneuropathy | G53.3*, G60.3, G61.8, G61.9, G61.0, G62.8, G62.9, G13.0*, G63.1*, G63.5* |
| N-autonomic neuropathy | G90.0, G99.1* |
| N-eye | H20, H35.3, H35.4, H35.0, H46 |
| **Conditions of the neuromuscular junction and muscle** |  |
| NM-Junction | G70.0,G71.1, G73.1* |
| Muscle | G72.4, G72.9, G73.2*, G73.7*, M33.2, M33.1, M33.9, M36.0*, M35.3 |
| Other myositis | H05.1, M60.8 |
| **Ménière’s disease** | H81.0 |
| **Circulatory conditions (not described as PNS)** | I00, I01, I02, I27.0, I42.0, I40.1, I40.8, I40.9, I51.4, I41.8* |
| **Asthma** | J45 |
| **Digestive conditions (not described as PNS)** | K11.8, K29.4, K31.8, K50, K51, K73.2, K74.3, K75.4, K85, K83.0, K90.0 |
| **Kidney disease** |  |
| R-Glomerulonephritis | N00 to N05 with .2 ending, N00 to N06 with .0 ending, N00 to N06 with .1 ending, N00 to N06 with .5 and .6 ending, N02 with ending .1, .2, .3, .5 and .8, N01 with ending .0 .1 .2 .3 .4 .5 .6 .7 .8 .9 |
| R-glomerular disorders | N08.8*, N08.5*, N08.1 |
| R-other | N12, N26 |
| **Dermatologic conditions** |  |
| D-bullous | H13.3*, L10, L12, L13.0 |
| Pruritus | D29 |
| D-alopecia | L63, L65.0, L66.8, L66.9 |
| D-papulosquamous | L83, L85.0, L85.1, L85.9 |
| Seborrheic keratosis | L82 |
| D-dermatoses | L98.2, L88 |
| Psoriasis | L40 |
| D-other | D76.3, K13.0, L68.1, L80, L81.4, L98.5 |
| **Rheumatic syndromes** |  |
| R-arthropaties | L62.0, M89.4, M02.3, M13.0, M36.1*, M65.8, M65.9, M68.8*, M72.9, M94.1 |
| R-Rheumatoid arthritis | M05, M06 |
| R-Autoimmune syndromes | L93, M32, L94.0, M34, M35.0, M35.1, M35.2, M35.8, M35.9, M45 |
| R-panniculitis | M35.4, M35.6, M79.3 |
| **Non system-specific conditions** |  |
| General fever | R50.8, R50.9 |
| Cachexia | R64 |
| Laboratory: abnormal level of other enzymes / raised antibody titer | R74.8, R76.0 |
